# Modelling mosquito infection at natural parasite densities identifies drugs targeting EF2, PI4K or ATP4 as key candidates for interrupting malaria transmission

**DOI:** 10.1038/s41598-017-16671-0

**Published:** 2017-12-15

**Authors:** Koen J. Dechering, Hans-Peter Duerr, Karin M. J. Koolen, Geert-Jan van Gemert, Teun Bousema, Jeremy Burrows, Didier Leroy, Robert W. Sauerwein

**Affiliations:** 1 TropIQ Health Sciences, Transistorweg 5-C02, 6534AT Nijmegen, The Netherlands; 2 Numerus Limited, Hans-Sahl-Straße 2, 72074 Tübingen, Germany; 3 Radboud University Medical Center, PO Box 9101, 6500 HB Nijmegen, The Netherlands; 4 Medicines for Malaria Venture, Route de Pré-Bois 20, 1215 Geneva, 15 Switzerland

## Abstract

Eradication of malaria requires a novel type of drug that blocks transmission from the human to the mosquito host, but selection of such a drug is hampered by a lack of translational models. Experimental mosquito infections yield infection intensities that are substantially higher than observed in natural infections and, as a consequence, underestimate the drug effect on the proportion of mosquitoes that become infected. Here we introduce a novel experimental and computational method to adequately describe drug efficacy at natural parasite densities. Parameters of a beta-binomial infection model were established and validated using a large number of experimental mosquito infections at different parasite densities. Analyses of 15 experimental and marketed drugs revealed a class-specific ability to block parasite transmission. Our results highlight the parasite’s elongation factor EF2, PI4 kinase and the ATP4 sodium channel as key targets for interruption of transmission, and compounds DDD107498 and KAE609 as most advanced drug candidates.

## Introduction

Since 2000, a global increase in the implementation of control measures has significantly decreased the burden of malaria^1^. With these successes in mind, global eradication seems feasible and the World Health Organization has put forward the vision of a world free of malaria with an intermediate milestone of 90% reduction in incidence and mortality rates by 2030^2^. This is a daunting task, as the disease currently continues to threaten half of the world’s population leading to 429,000 deaths annually^3^. A complicating factor is that current medication does not effectively clear the parasite stages that are infectious to the mosquito host, and patients cured from clinical disease may continue to transmit the pathogen for several weeks^4^. Achievement of the global eradication goal is critically dependent on the generation of novel intervention tools that target the transmission reservoir in the human and mosquito host^5^. The past decade has seen a renaissance in malaria drug discovery^6,7^. The gold standard for selection of transmission-blocking molecules from the emerging portfolio of candidate molecules is the Standard Membrane Feeding Assay (SMFA), where suspensions of cultured *P. falciparum* gametocytes are exposed to test compounds and fed to *Anopheles* mosquitoes. Approximately one week later the yield of infection is determined by counting the number of oocysts per infected mosquito (infection intensity) and the proportion of mosquitoes that carries at least one oocyst (infection prevalence)^8,9^. A drawback of the SMFA is that laboratory infections show 5 to 10-fold higher oocyst loads per mosquito than observed in natural infections, where parasite densities average at ~3 oocysts/midgut^10–14^. As a result, the effect on infection prevalence is underestimated^15^. For example, a drug that reduces the number of oocysts by 50% from 100 to 50 oocysts per midgut will hardly affect infection prevalence whereas an equivalent fractional reduction from 1 to 0.5 oocyst will significantly impact infection prevalence^16,17^. This problem that drug effects on infection prevalence are very sensitive to changes in baseline infection intensity has been recognized before, and many studies have limited their analyses to reports on oocyst intensities^14,15,18^. There is an obvious divide between this outcome and the measure that is most relevant for public health, which is the reduction in the number of potentially infectious bites received by a person over a given time period. This Entomological Inoculation Rate is determined by the number of parasite-positive mosquitoes and their parasite loads^19^. Efficacy of a transmission-blocking drug would ultimately depend on reducing the proportion of infectious mosquitoes and the related force of infection experienced by the human population^16,17^. It is, therefore, critical to evaluate candidate drugs for their ability to reduce infection prevalence at low infection intensities as observed in natural infections. In the present study, we set out to develop an experimental and computational strategy to assess compound effects on the infection prevalence at naturally occurring infection intensities. Our results show that infection prevalence can be robustly derived from modeling the infection intensity on basis of a beta binomial distribution of oocyst numbers. We have used this model to systematically evaluate the transmission-blocking effects of a set of mechanistically distinct antimalarials in preclinical and clinical development, and, for comparison, a set of marketed drugs.

## Results

### Dose-dependent reduction of oocyst intensities

A dataset was generated by testing the dose-dependent effect of 15 different marketed and experimental antimalarial compounds in the SMFA. To this end, *P. falciparum* NF54 gametocytes were exposed to serial dilutions of compounds for 24 hours prior to feeding to *Anopheles stephensi* mosquitoes. The test compound remained present during feeding, mimicking a situation where a mosquito would feed on a gametocyte carrier with circulating drug levels. All compounds were tested at 9 different dilutions in duplicate. The baseline infection rate was determined by including 2 vehicle control feeders in every experimental run. Six to eight days post infection, mosquitoes were dissected to determine the oocyst intensities. Data for compounds DHA, KDU691, DDD107498 have been published in a different form previously, but were re-analyzed here^8,20,21^. A total of 8102 mosquitoes from 428 experimental feeds were analyzed. Parasite densities in the mosquito ranged from 0 to 126 oocysts per midgut, with a median of 9 and a mean of 13.8 oocysts/midgut for mosquitoes from the control feeders.

**Figure 1:**
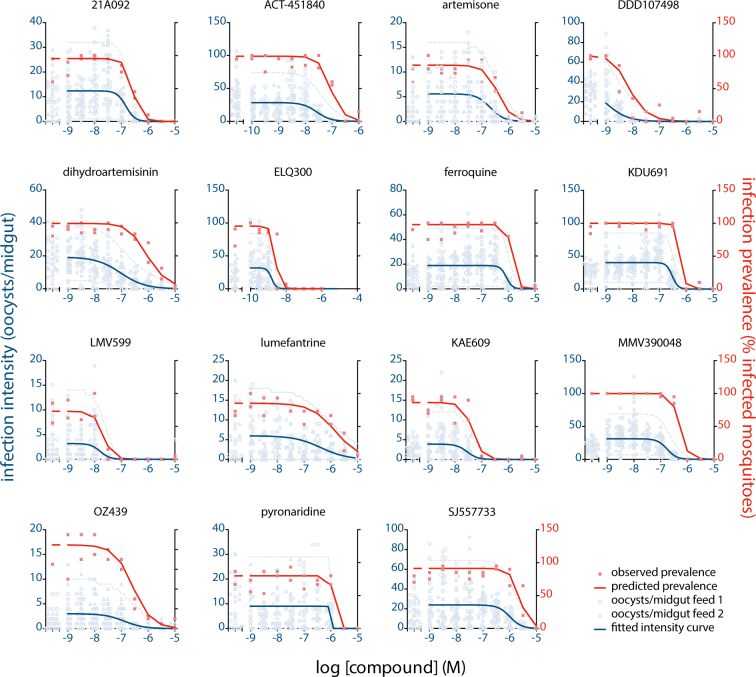
Dose dependent reduction of infection intensity and infection prevalence. The figure shows oocyst intensities in individual mosquitoes from two independent feeds per drug concentration tested (open symbols). The left x-axis shows the infection intensity in the vehicle control feeders. The blue line shows the fit from the BBD model. The light blue dashed lines show the region of tolerance containing 95% of the observed oocyst intensities. The solid red symbols indicate the experimentally determined infection prevalence from the proportion of infected mosquitoes per feed. The solid red line indicates the infection prevalence predicted by the BBD model based on the fitted dispersion of oocysts.

The distribution of oocyst numbers in mosquitoes fed on a single feeder was modeled by a beta binomial distribution (BBD). The BBD model addresses the over-dispersion of oocyst intensities due to high numbers of uninfected mosquitoes. It is an alternative for negative binomial distribution models described previously but alleviates the need for zero-inflation strategies to explain the observed distribution of oocyst counts^15,18^. To describe dose-response relationships, mean oocyst intensities *μ* as a function of drug concentration *c* were fitted to a Hill function according to equation:

1 $$\mu (c)=\frac{{\mu }_{0}}{1+{10}^{s(-pIC{50}_{intensity}-lo{g}_{10}(c))}}$$

whereby ${\mu }_{0}$ is the baseline mean infection intensity, pIC50_intensity_ is the negative log of the concentration at the inflection point, and *s* is the Hill slope of the dose-response profile. Dispersion of oocyst distributions was parameterized by the variance to mean ratio (VMR), which describes the degree of over-dispersion of a distribution and is a determinant of prevalence of infection (large VMRs indicate over-dispersed oocyst distributions of which the prevalence is lower compared to less over-dispersed distributions). Maximum Likelihood Estimation was used to find the best fit to the model parameters. Figure 1 shows the dispersion of oocyst counts and the fitted dose response curves. The data indicate that compounds DDD107498 and ELQ300 were most potent in reducing oocyst intensities, with pIC50_intensity_ of 9.0 and 8.8, respectively. These potencies correspond with their activities against asexual blood stage parasites responsible for clinical malaria (Table 1). In contrast, compounds dihydroartemisin and artemisone from the class of endoperoxides, used as first line malaria treatment, showed 100-fold lower potency in the SMFA than observed in asexual replication assays. Likewise, ACT451840, PA21A092, SJ55773, pyronaridine, ferroquine and lumefantrine showed relatively poor transmission blocking activity.

**Table 1:** Overview of model estimates from experimental data.

Compound	Target/mechanism	baseline infection intensity (95% CI)	VMR	Hill slope (95% CI)	pIC50 intensity (95% CI)	pIC50 prevalence (95% CI)	normalized pIC50 prevalence_, µ0=3_	pIC50 asexual asexual bloodstage	human dose (mg)	pC_avg_, 0–24 hrs	ΔpIC50 prevalence_,µ0=3_;pC_avg_	ΔpIC50asexuals;prevalence_,µ0=3_
ELQ300	BC1 / ATP production	31.82 (27.5; 36.2)	14.50	−3.44 (−4.5; −2.4)	8.81 (8.7; 8.9)	8.58 (8.5; 8.6)	8.69	8.32^33^				−0.4
DDD107498	eEF2 / protein synthesis	37.08 (28.7; 45.4)	14.80	−1.07 (−1.3; −0.9)	9 (8.8; 9.2)	8.12 (8.1; 8.1)	8.63	9.00^20^				0.4
KDU691	PI4K / membrane trafficking	40.17 (37.6; 42.7)	9.46	−4.05 (−4.9; −3.2)	6.52 (6.5; 6.6)	6.24 (6.2; 6.3)	6.42	7.24^21^				0.8
MMV390048	PI4K / membrane trafficking	31.28 (28.9; 33.6)	8.42	−2.42 (−3.1; −1.7)	6.76 (6.7; 6.9)	6.32 (6.3; 6.3)	6.60	7.55^34^	80(☥)	6.29	0.30	1.0
LMV599	PI4K / membrane trafficking	3.21 (2.1; 4.3)	4.75	−2.41 (−3.4; −1.5)	7.79 (7.7; 7.9)	7.81 (7.7; 7.9)	7.62	8.72(*)				1.1
PA21A092	ATP4 / sodium homeostasis	12.4 (11.2; 13.6)	2.29	−2.61 (−3.3; −1.9)	6.83 (6.7; 6.9)	6.57 (6.5; 6.6)	6.68	8.30^35^				1.6
SJ557733	ATP4 / sodium homeostasis	23.88 (21.4; 26.3)	13.61	−1.99 (−2.6; −1.4)	5.99 (5.8; 6.1)	5.67 (5.6; 5.7)	5.79	7.52^36^				1.7
KAE609	ATP4 / sodium homeostasis	3.9 (3.2; 4.6)	2.79	−2.22 (−3; −1.5)	7.54 (7.4; 7.7)	7.4 (7.3; 7.5)	7.36	9.3^37^	75^29^	6.00	1.35	1.9
ACT451840	PfMDR1 / transporter	28.53 (25.8; 31.2)	12.17	−1.67 (−2; −1.3)	7.57 (7.5; 7.7)	7.06 (7; 7.1)	7.33	9.40^38^				2.1
OZ439	heme metabolism	3.01 (2.4; 3.6)	2.40	−1.19 (−1.5; −0.9)	6.89 (6.7; 7.1)	6.78 (6.6; 6.9)	6.55	8.72^39^	800^40^	6.15	0.40	2.2
pyronaridine	heme metabolism	9.03 (7.8; 10.3)	6.85	−18.27 (na;na)	6 (na; na)	5.98 (na; na)	5.98	8.31^25^	180^41^	6.49	−0.51	2.3
DHA	heme metabolism	19.26 (17.5; 21)	4.07	−0.95 (−1.1; −0.8)	7.03 (6.8; 7.2)	5.98 (6; 6)	6.61	8.96^25^	480^42^	6.91	−0.30	2.3
lumefantrine	heme metabolism	5.99 (4.8; 7.2)	3.69	−0.88 (−1.4; −0.4)	6.37 (5.8; 6.9)	5.96 (5.7; 6.1)	5.92	8.55^25^	960^43^	5.02	0.89	2.6
artemisone	heme metabolism	5.55 (4.7; 6.4)	3.23	−1.69 (−2.2; −1.2)	6.68 (6.5; 6.9)	6.47 (6.4; 6.6)	6.45	9.10^25^				2.6
ferroquine	heme metabolism	18.93 (17.4; 20.5)	8.09	−3.95 (−5.2; −2.7)	6.07 (6; 6.1)	5.87 (5.8; 5.9)	5.97	8.72^44^				2.8

Columns list the following parameter estimates (for parameters see Methods, Statistical analyses): 1) Baseline infection intensity (*μ*_0_): average number of oocysts per mosquito midgut for the experiments analysed here. 2) VMR: variance to mean ratio as estimated by the BBD model. 3) Hill slope (*s*): slope of the Hill function. 4) pIC50_intensity_: IC50 of infection intensity. 5) pIC50_prevalence_: IC50 of oocyst prevalence (see Methods, logistic regression). 6) normalized pIC50_prevalence, µ0=3_: pIC50 for infection prevalence normalized on a baseline infection intensity of 3 oocysts per midgut according to formula (2), allowing for comparisons between compounds and experiments. In addition, the table lists pIC50 values as reported for activity against the asexual blood stages. To compare pIC50_prevalence_ values to human exposure data the table lists previously published plasma concentration as a pC_avg_ (−log C_avg_) for the first 24 hours (pC_avg,0–24hrs_) following administration of the dose indicated in the column ‘human dose’. The column next to the pC_avg_ values lists the difference between the normalized pIC50_prevalence_ value and the pC_avg_ value, to indicate the level of exposure above the transmission-blocking pIC50 value. Lastly, the table lists the difference between the asexual blood stage pIC50 and normalized pIC50_prevalence_. This value indicates the gap between the therapeutic and the transmission-blocking activity. The compounds are sorted from smallest to largest gap from top to bottom. CI: confidence interval. na: confidence interval not computed as the model did not fully converge at very steep Hill slopes. *Bryan Yeung, personal communication, ☥DL, unpublished data.

### Drug effects on oocyst prevalence

**Figure 2:**
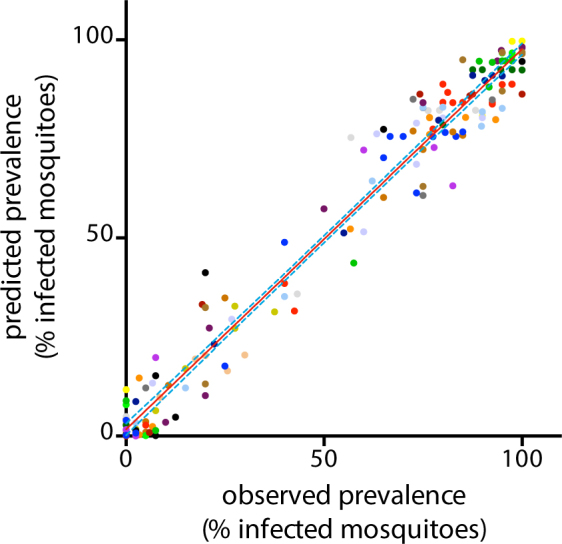
Validation of the model-predicted infection prevalence. The figure shows the correlation between observed prevalence and predicted prevalence for all experimental feeds. Identical colors indicate data from one dose response experiment. The red solid line indicates the regression line determined by linear regression using a least-squares method to find the best fit (R2 = 97%). The dashed blue lines indicate the 95% confidence intervals of the regression line.

Previous work has shown that the relative reduction in oocyst numbers is independent of the baseline infection intensity^15,18^. Thus, the pIC50 estimate on infection intensity will give similar results for experiments with high baseline infection intensities and experiments with much lower densities. In contrast, the relative reduction in oocyst prevalence, or the proportion of mosquitoes that carry at least one oocyst, is heavily dependent on the intensity of the infection. To further investigate this relationship we assessed the performance of the BBD model in estimating oocyst prevalence. The prevalence estimates obtained from modeling the intensity data showed an excellent correlation with observed prevalence in experimental feeds (R^2^ = 97%, Fig. 2). To further visualize the goodness-of-fit across the range of drug concentrations tested, the predicted prevalence values were plotted in the dose response plots in Fig. 1. The results show a near perfect fit with the experimentally determined prevalence data. To compare the experimental reduction in infection intensity and oocyst prevalence, pIC50_prevalence_ values were derived from the inflection points of the modeled prevalence curves. In general, the prevalence dose response curves show a right-shift in comparison to the intensity curves, and for all compounds the pIC50_prevalence_ values were lower than the pIC50_intensity_ values (Table 1). Notably, experiments with dihydroartemisinin and DDD107498 showed a large discrepancy between pIC50_intensity_ and pIC50_prevalence_. This relates to the high baseline infection intensity and shallow Hill slopes of these compounds as will be explained below.

### Prevalence as a function of intensity

**Figure 3:**
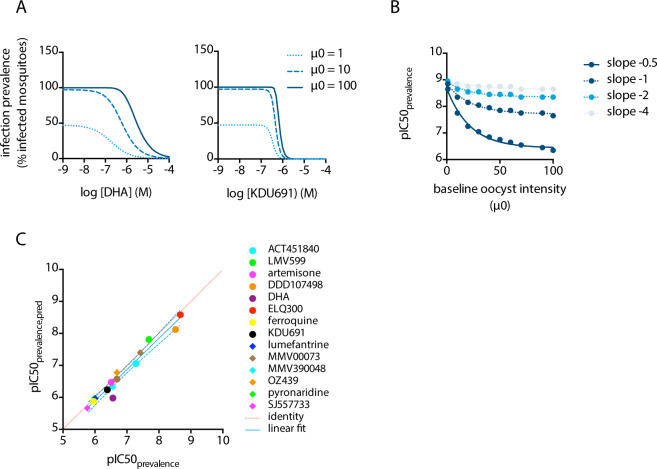
(**A**) Simulated dose response curves for DHA and KDU691 at baseline oocyst intensities (µ0) of 1, 10 and 100 oocysts/midgut. (**B**) relationship between the pIC50 of infection prevalence (pIC50_prevalence_) and the baseline infection intensity at different Hill slopes. Symbols indicate simulated data from the BBD model, the solid lines represent the results from a non-linear regression analysis using least squares to find the best fit. The simulations were run using a fixed value of 9 for the pIC50_intensity_ parameter. The results indicate that the pIC50_prevalence_ deviates from the pIC50_intensity_ with increasing baseline infection intensities and shallower hill slopes. (**C**) correlation between observed and predicted pIC50 for infection prevalence. The figure compares the pIC50_prevalence_ values derived by non-linear regression of the data presented in Fig. 1 to the predicted values (pIC50_prevalence,pred_) calculated according to formula (2). The blue solid line indicates the regression line determined by linear regression using a least-squares method to find the best fit (R2 = 96%), with the dashed blue lines indicating the 95% confidence interval. The dashed red line indicates the identity (y = x).

Figures 1 and 2 indicate that infection prevalence is well predicted by the BBD model. The baseline infection intensity for these experiments was 13.8 oocysts/midgut on average, which is much higher than generally observed in natural infections with 2–3 oocysts on average^10–12^. Therefore, we adapted the BBD model to simulate prevalence data at natural infection intensities. In keeping with previous reports^22,23^, we observed that the variance was proportional to the mean infection intensity (Fig. S1). Incorporating this function into the model allowed the simulation of compound efficacy at different infection intensities. The results of such simulations show that the efficacy in reducing infection prevalence increases with lower infection intensities, leading to an increase in the prevalence pIC50. This shift in pIC50 is more extreme with compounds that show low Hill coefficients (shallow curves). Figure 3A provides simulated examples for compounds DHA and KDU691 that showed shallow and steep Hill slopes, respectively. For DHA, the inflection point of the prevalence curve shifts 16-fold (pIC50 5.5 to 6.7) when baseline oocyst intensities drop from 100 to 1 oocysts/midgut. In contrast, KDU691 is less sensitive to changes in parasite exposure and its inflection point shifts 2-fold (pIC50 6.2 to 6.5) when baseline oocyst intensities decrease from 100 to 1 oocysts/midgut. Figure 3B presents a more generalized description of these findings. It shows the simulated pIC50 of infection prevalence as a function of infection intensity for hypothetical compounds with pIC50_intensity_ of 9, and Hill coefficients ranging from −0.5 to −4. The simulations show that an increase in infection intensity results in a decrease in prevalence pIC50. This effect is more pronounced with more shallow Hill slopes.

Next, we derived the function describing the data depicted in Fig. 3B by logistic regression using a least squares method to find the best fit (Fig. S2). The results showed that the pIC50 prevalence at any given infection intensity can be described by the following equation:

2 $$\,pIC{50}_{prevalence,pred}=\,pIC{50}_{intensity}+\frac{0.114+0.595\,lo{g}_{10}({\mu }_{0,target})}{{S}_{intensity}}$$

where S_intensity_ relates to the experimentally derived Hill slope of the intensity dose response curve and µ_0,target_ is the baseline infection intensity for which the IC50_prevalence_ needs to be predicted. To evaluate whether this function accurately predicts experimental data, we plotted pIC50_prevalence,pred_ calculated according to (2) against the pIC50_prevalence_ values derived from the dose response curves depicted in Fig. 1. The results show an excellent correlation between the fitted and calculated pIC50 values (Fig. 3C).

### Drug efficacy at low infection intensities

Baseline oocyst intensities are known to vary greatly between individual SMFAs, even if highly standardized protocols are used^8,14,15,24^. In order to allow a comparison between data from different experiments we used formula (2) to calculate a normalized pIC50_prevalence, µ0=3_ where we used a baseline infection intensity of 3 as representative for natural infections. We subsequently ranked compounds according to the difference observed between the normalized prevalence pIC50 and the pIC50 as observed against asexual blood stages (Table 1). The larger the difference, the less likely a compound will reduce transmission at concentrations that are relevant to its clinical efficacy in reducing asexual blood stage parasitemia. The resulting rank order deviates significantly from the order obtained by simply ranking compounds on basis of their asexual blood stage activity, and from the order obtained by ranking compounds on their pIC50_intensity_ (Table S1). This indicates the importance of a proper assessment of transmission-blocking activity next to the determination of asexual blood stage activity. For a number of experimental antimalarials with available human pharmacokinetics data, we calculated the difference between the average plasma exposure in the first 24 hours after dosing (in -log M), and the pIC50_prevalence_. This value shows, on a log scale, the multiple of the IC50_prevalence_ that on average is achieved following the anticipated human dose. For comparison, we included data for marketed compounds lumefantrine, dihydroartemisinin and pyronaridine. The latter two compounds showed a negative log difference between the average plasma concentration and the pIC50_prevalence_, indicating that the human exposure does not reach levels that are likely to block transmission. For lumefantrine at a dose of 960 mg, the reported C_avg_ levels were 7.8-fold above the IC50_prevalence_. For the experimental antimalarials described here, OZ439 and MMV390048 showed a modest C_avg_ exposure above the IC50_prevalence_, with a multiple of 2.5 and 2.0 (log 0.4 and 0.3), respectively. In contrast, KAE609 shows within the first 24 hours following a total dose of 75 mg average plasma concentrations that are 22-fold above the IC50_prevalence_. This indicates a clear potential for achieving blockage of transmission at curative doses.

## Discussion

Identification of the key pathways and candidate drugs for interrupting malaria transmission is essential to the implementation of strategies for malaria eradication. The methodology presented here provides a path forward for triaging and prioritizing candidate drugs according to their ability to reduce mosquito infection rates. Modelling the distribution of oocyst numbers by means of a beta binomial distribution showed excellent properties with respect to model parameterization and goodness-of-fit. Dose response relationships could be described by a parsimonious model with only four parameters, the pIC50_intensity_, Hill slope, baseline infection intensity and the VMR. This is in line with previous work that described the relationship between infection intensity and prevalence and highlighted that prevalence is determined by infection intensity and the degree of overdispersion of oocyst counts^15,18^. Our results indicate that VMR is a simple function of the infection intensity, and experimental data from high intensity infections may be used to predict infection outcomes at lower parasite exposures. Where inter-SMFA comparison of drug effects on infection prevalence was previously not feasible due to differences in baseline infection intensities, the introduction of a normalized pIC50 value resolves this issue and makes experimental data comparable. Using prevalence pIC50 values normalized to a baseline infection intensity of 3, which is in line with values observed in natural infections, we ranked 15 compounds from the global portfolio of marketed and experimental antimalarials according to magnitude of the gap between their asexual blood stage and transmission-blocking potency. The resulting rank order deviates from a rank order that would be based on pIC50_prevalence_ values prior to normalisation, as for compounds like DHA and DDD107498 these values were severely underestimated as a result of the high baseline infection intensity in these experiments. Our ranked list reveals similar transmission-blocking properties of compounds that share the same mechanism of action. The results are in agreement with previous observations that compounds selected against the asexual blood stages in generally have a weaker activity against the transmission stages of the parasite^13^. The exception is provided by compound ELQ300 that showed a more potent transmission stage activity than asexual blood stage activity. Its mechanism of action depends on inhibition of the bc1 complex in the mitochondrial electron transport chain. This pathway is essential for replication of asexual blood stage parasites and sporogonic development in the mosquito, but dispensable for gametocyte maturation and gamete formation^13,25^. For a transmission-blocking drug, a sporontocidal mode of action limited to the mosquito stages of the parasites is less favoured than a gametocytocidal activity against parasites in the human host. Maturation of gametocytes takes around 10 days, and a drug that does not kill gametocytes but acts in the mosquito midgut to block onward parasite progression should have a very long circulating half-life in order to interrupt the cycle. In this respect, eEF2 inhibitor DDD107498 is a more attractive development candidate for reducing malaria transmission as its mode of inhibition of transmission depends on a gametocytocidal activity, combined with a weaker sporontocidal action^20^. Our data indicate it is equipotent in reducing infection prevalence and inhibiting replication of asexual blood stage parasites. Indeed, at the minimum dose that fully cleared asexual blood stage parasites, it showed a 91% reduction in infection prevalence of mosquitoes fed on drug-treated mice^20^. These mice were infected with *P. berghei* parasites, resulting in an average infection intensity of 36.9 oocysts per midgut. At this parasite exposure, the experiment was likely to underestimate the efficacy on reducing prevalence for reasons described here. Further progression of DDD107498 as an antimalarial agent depends on the outcome of phase I safety studies, which are expected in the course of 2017. Further down the list of ranked compounds, PI4kinase inhibitors KDU691, LMV599 and MMV390048 all showed a 6 to 13-fold (log 0.8–1.1) difference between their asexual blood stage and transmission stage potencies. For ATP4 channel blockers PA21A092, KAE609 and SJ557733, this difference ranged from 40 to 80-fold (log 1.6–1.9). The other compounds in our ranked list showed a greater than 100-fold difference between their asexual blood stage potency and transmission-blocking potency. Their potential application as a transmission-blocking drug seems unlikely, unless the gametocytocidal activity is driven by a short exposure to a peak concentration instead of a prolonged exposure to a lower concentration. In our SMFA experiments, parasites were exposed to drug for 24 hours. For a number of compounds that have human exposure data available, we compared the average 24-hour post-dosing exposure to the mosquito infection prevalence IC50, as a rough approximation to whether a transmission-blocking effect may be expected. For lumefantrine, that is used as part of artemisinin-based combination therapies (ACT), the C_avg0–24_ is 7.8-fold (log 0.89) above the infection prevalence IC50, following a loading dose of 960 mg on the first day of treatment, This suggests that some reduction on transmission may be achieved. Few studies have investigated mosquito infection after treatment with malaria drugs. A study in the Gambia showed a greater effect of artemether/lumefantrine than of chloroquine plus sulfadoxine/pyrimethamine on parasite transmission of human gametocyte carriers to mosquitoes. Compared to other ACTs, artemether lumefantrine seems to exert the strongest transmission-reducing effect, suggesting that indeed the lumefantrine component contributes to a reduction in the number of infected mosquitoes^26–28^. The artemisinin component has weak effects on transmission, and our data indicate that DHA, used in combination with piperaquine, does not reach plasma exposure above the transmission-blocking IC50.

For the PI4K inhibitor MMV390048, human pharmacokinetic studies showed that average plasma levels in the 24 hours following a human dose of 80 mg are 2-fold above the IC50_prevalence, µ0=3,_ which may lead to a partial reduction in parasite transmission *in vivo*. In spite of its lower transmission blocking potency, the profile of ATP4 inhibitor KAE609 looks more attractive. Average plasma levels within 24 hours following a human dose of 75 mg are 22-fold above the IC50_prevalence, µ0=3_, with remaining plasma levels of approximately 600 nM after 24 hours^29^_._ Based on the characteristics of the dose response relationship described here, the 99% inhibitory concentration for reducing mosquito infection prevalence under natural infection intensities is estimated at 346 nM. Thus, a human dose of 75 mg is likely to lead to full sterilization of circulating gametocytes and blockage of mosquito infection.

Ultimately, prediction of a human effective dose depends on an understanding of the changes in *in vivo* drug levels in time and its effect on infectivity of the circulating gametocytes. This would benefit from a rodent transmission model that would allow the determination of an *in vivo* ‘minimum sterilizing concentration’, in analogy with the mimimum parasiticidal concentration that is established for asexual blood-stage active drugs, where it defines the minimum *in vivo* concentration that achieves the maximum parasiticidal effect^30^. A rodent malaria transmission model has been proposed on basis of infection with *P. berghei* parasites^31^. Since this is a different species of *Plasmodium* its value for human malaria may be limited. Furthermore, oocyst intensities in mosquitoes infected through this model are very high (>40 on average) and the model will severely underpredict drug efficacy on infection prevalence for the reasons discussed here. Further development of promising molecules such as DD107498 would require a humanized rodent transmission model and, ultimately, a controlled human transmission model^32^.

## Methods

### Standard Membrane Feeding Assay

Standard Membrane Feeding Assays were performed as described previously^8^. Briefly, compounds were serially diluted in DMSO and subsequently in RPMI1640 medium supplemented with 10% human type A serum (Sanquin, The Netherlands). Diluted compounds were combined with *P. falciparum* NF54 stage V gametocytes and incubated for 24 hours at 37 °C in an Eppendorf tube. Following pre-incubation, parasites were pelleted by centrifugation and resuspended in human red blood cells in human type A serum to a hematocrit of 56%. Freshly diluted compound was added to the serum. The blood meal was fed to *Anopheles stephensi* mosquitoes using mini membrane feeders covered with a Parafilm membrane. Twenty-two feeders were used simultaneously, allowing analysis of nine compound dilutions and two positive (10 µM dihydroartemisinin) and two negative (0.1% DMSO) controls in a single experimental run. Non- and partially-fed mosquitoes were removed from the cages within 4 hours post feeding. Mosquitoes were maintained at 26 °C and 70–80% humidity. Six to eight days later, midguts were dissected, stained with 1% mercurochrome and mounted on glass slides for microscopic examination of the number of oocysts per midgut (oocyst intensities). Oocyst prevalence was expressed as the percentage of mosquitoes from a single cage that carried at least one oocyst.

### Statistical analyses

Oocyst counts were modelled by the beta binomial distribution (BBD) given by $$P(k|n,\,\alpha ,\,\beta )=(\begin{array}{c}n\\ k\end{array})\frac{B(k+\alpha ,\,\,n-k+\beta )}{B(\alpha ,\,\beta )}$$, whereby *k* is the number of oocysts in a mosquito, *n* is the maximum possible number of oocysts in a mosquito, and *B(α,β)* is the beta function. Based on historic experimental laboratory infection data, we assumed that maximally *n = *300 oocysts can be found in a mosquito (observed maximum in the current dataset: *n = *126). A sensitivity analysis based on *n* = 500 showed that estimates do almost not depend on *n* (results not shown). With $p=\frac{\alpha }{\alpha +\beta }$ as the proportion of *n* oocysts, and dispersion parameter $\delta =\frac{1}{(\alpha +\beta +1)}$, expectation and variance of the *BBD(n, α, β*) are $\mu =np$ and ${\sigma }^{2}=np(1-p)[1+\delta (n-1)]$. For purposes of biological interpretation we parameterize the BBD by *p* and $VMR=\frac{{\sigma }^{2}}{\mu }=(1-p)[1+\delta (n-1)]$, yielding *BBD(n, p, VMR)*: $$P(k|n,p,\delta )=(\begin{array}{c}n\\ k\end{array})\frac{B(k+p(\frac{1}{\delta }-1),\,n-k+(1-p)(\frac{1}{\delta }-1))}{B(p(\frac{1}{\delta }-1),(1-p)(\frac{1}{\delta }-1))}$$, whereby $\delta =\frac{\frac{VMR}{(1-p)}-1}{n-1}=\frac{1}{(\alpha +\beta +1)}$.

The mean oocyst number at a particular drug concentration, *μ(c)*, was modeled by a Hill function of type $p(c)=\frac{{p}_{0}}{1+{10}^{s(lo{g}_{10}(IC50)-c)}}$, whereby *c = log*_*10*_ *(molar drug concentration), μ*_*0*_ = *p*_*0*_ *n* is the baseline mean oocyst number for *c* = 0*, s* is the Hill slope and *IC*_50_ is the molar concentration at the inflection point of the Hill curve. The −log *IC*_*50*_ is referred to as the pIC50_intensity_ throughout the text. Parameter vector $\overrightarrow{{\theta }_{E}}=\{{\mu }_{0},VMR,I{C}_{{50}},\,s,\}$ has been estimated by Maximum Likelihood (ML) for each experiment *E* based on the *BBD(n, p(c), VMR)*. The corresponding 95% confidence intervals (CI) were derived from the corresponding Profile likelihood. The probability that mosquitoes do not have any oocysts is $P(0|n,p(c),\delta (c))=\frac{B(p(c)(1/(\delta (c))-1),\,n+(1-p(c))(1/(\delta (c))-1))}{B(p(c)(1/(\delta (c))-1),(1-p(c))(1/(\delta (c))-1))}$; the proportion of infected mosquitoes is then $P(c)=1-P(0|n,p(c),\delta (c))$. Estimates and 95% CI for the pIC50_prevalence_ have been derived from a constraint optimization given ML-estimates ${\overrightarrow{\theta }}_{E}=\{{\hat{\mu }}_{0},VMR,I{\hat{C}}_{50},\,\hat{s},\}$ and their 95% CI. Since the proportion of infected mosquitoes is not a model parameter, but a model outcome, corresponding estimates were derived at the limits of the Profile likelihood of ${\hat{\theta }}_{E}$.

In order to simulate low parasite exposure data from high intensity experiments, and vice versa, the baseline *VMR* was approximated by $Lo{g}_{10}({\sigma }^{2})=a+{s}^{(Lo{g}_{10}(\mu )-b)}$ as shown in Figure S1. Resulting estimates were *a* = −12.95 and *b* = −25.91 and *s* = 1.105. The baseline VMR is then given by $VM{R}_{0}={{\sigma }^{2}}_{0}/{\mu }_{0}$.

Using the BBD model and above VMR_0_ estimate, infection prevalence pIC50 values were simulated for a range of baseline oocyst intensities from 0–100 and Hill slopes from −0.5–5.0 for a hypothetical compound with an pIC50 on infection intensity of 9. The simulated pIC50_prevalence_ decreased linearly with the Log-transformed baseline mean infection intensity and was fitted by $pIC{50}_{prevalence,pred}=pIC{50}_{intensity}+{a}_{1}/s+({b}_{1}+{b}_{2}/s)\,lo{g}_{10}({\mu }_{0})$. Least squares estimation suggested ${b}_{1}$ as not significantly different from zero, so that the model could be reduced to $pIC{50}_{prevalence,pred}=\,pIC{50}_{intensity}+\frac{{a}_{1}+{b}_{2}\,lo{g}_{10}({\mu }_{0})}{s}$. Estimates (95% CI) from logistic regression were, *a*_1_ = 0.114 (0.090–0.138), and *b*_2_= 0.595 (0.580–0.610).

Compound effects on parasite transmission were compared to previously reported activities against asexual blood stage parasites. To this end, the −logIC50 (pIC50) was derived from IC50 values previously published as listed in Table 1. To compare pIC50_prevalence_ to circulating drug levels of humans exposed to MMV390048, KAE609, OZ439, pyronaridine, dihydroartemisinin or lumefantrine, average drug levels were calculated based on published data and dosages indicated in Table 1. To this end, the published area under the curve values for the first 24 hours following the first dose were divided by 24 to give the average concentration C_avg_. This value was log transformed and the sign was inverted to yield a pC_avg_.

## Electronic supplementary material

Supplementary Information
