# Supplementary material for: Modelling mosquito infection at natural parasite densities identifies drugs targeting EF2, PI4K or ATP4 as key candidates for interrupting malaria transmission
Source: Sci Rep. 2017 Dec 15;7:17680. doi: 10.1038/s41598-017-16671-0 (PMC5732164; doi:10.1038/s41598-017-16671-0)
Supplement: Supplementary file 1 — Supplementary Information [file 41598_2017_16671_MOESM1_ESM.pdf]

Modelling mosquito infection at natural parasite densities identifies drugs targeting EF2, PI4K or ATP4 as key candidates for interrupting malaria transmission

Authors: Koen J. Dechering[1,*,†], Hans-Peter Duerr[2,†], Karin M.J. Koolen[1], Geert-Jan van Gemert[3], Teun Bousema[3], Jeremy Burrows[4], Didier Leroy[4] , Robert W. Sauerwein[1,3]

Affiliations:

[1]TropIQ Health Sciences, Transistorweg 5-C02, 6534AT, Nijmegen, The Netherlands

[2]Numerus Limited, Hans-Sahl-Straße 2, 72074, Tübingen, Germany

[3]Radboud University Medical Center, PO Box 9101, 6500 HB Nijmegen, The Netherlands

[4]Medicines for Malaria Venture, Route de Pré-Bois 20, 1215 Geneva 15, Switzerland

[†]These authors contributed equally

Supplementary Information

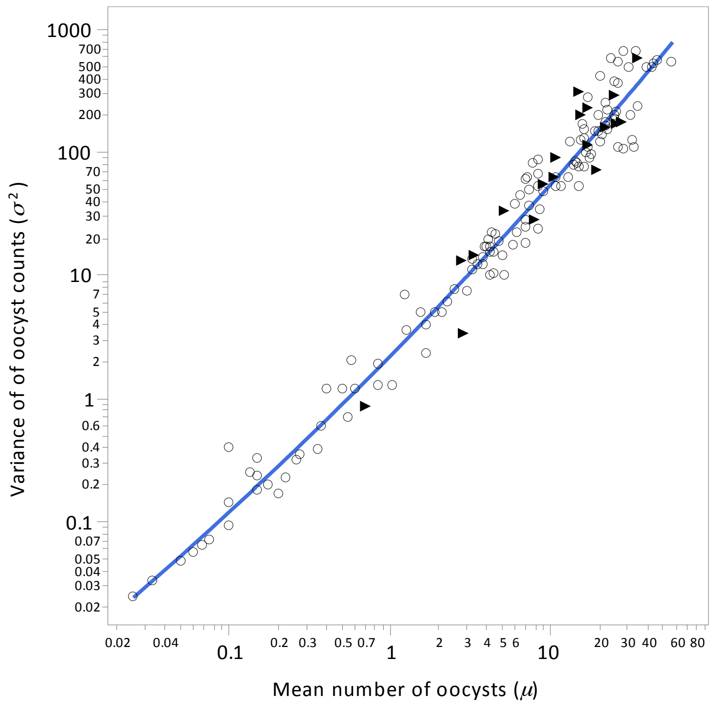

Fig. S1. Variance of oocyst counts as a function of the mean number of oocysts. The graphs shows mean oocyst counts and associated variance for all feeder data. DMSO controls are shown as filled triangles. The fitted curve has the form $Log_{10}(\sigma^2) = a + s^{(Log_{10}(\mu) - b)}$, with a = -12.95, b = -25.91 and s = 1.105.

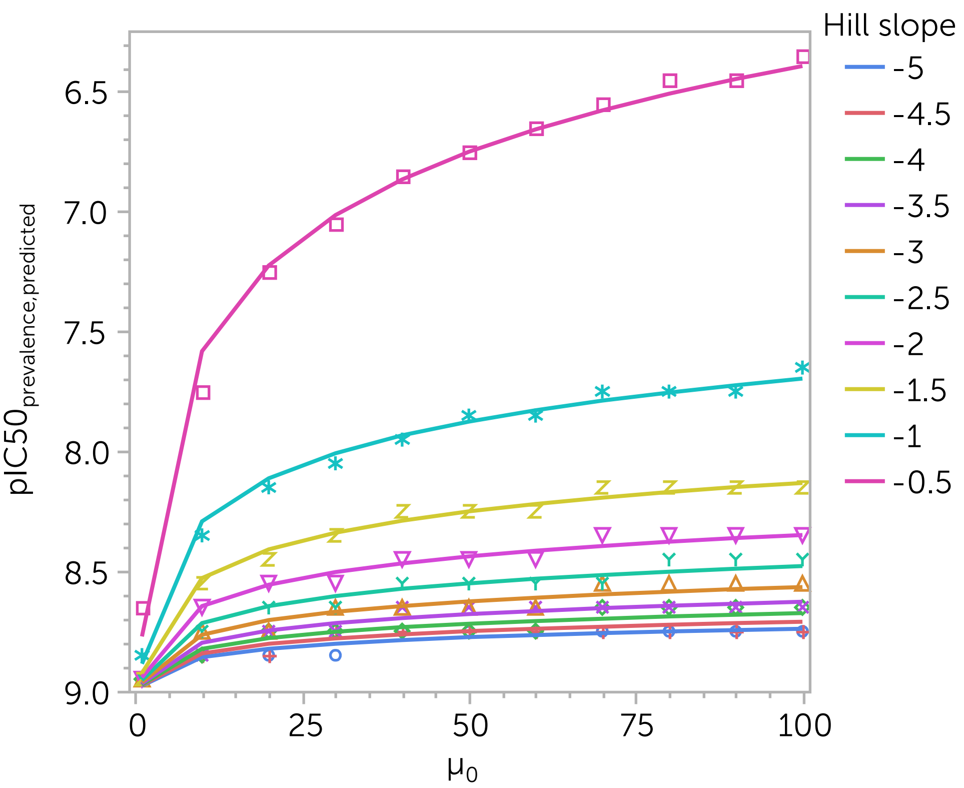

Fig. S2. Simulated pIC50$_{prevalence}$ data for a range of infection intensities (μ0) and Hill slopes (s) at a fixed pIC50$_{intensity}$ = 9. Symbols indicate simulated data from the BBD model, the solid lines indicate fitted model $pIC50_{prevalence,pred} = pIC50_{intensity} + \frac{a_1 + b_2\ log_{10}(\mu_0)}{s}$ where a_1= 0.114 and b_2= -0.595.

Compound	pIC50 asexuals	pIC50 intensity	pIC50 prevalence	pIC50 prevalence, normalized	ΔpIC50asexuals ,normalized prevalence
ACT451840	1	4	5	5	9
KAE609	2	5	4	4	8
artemisone	3	10	8	10	14
DDD107498	4	1	2	2	2
DHA	5	6	11	7	12
ferroquine	6	13	14	13	15
LMV599	7	3	3	3	5
OZ439	8	7	6	9	10
lumefantrine	9	12	13	14	13
ELQ300	10	2	1	1	1
pyronaridin	11	14	12	12	11
21A092	12	8	7	6	6
MMV390048	13	9	9	8	4
SJ557733	14	15	15	15	7
KDU691	15	11	10	11	3

Table S1. Rank order of compounds based on parameters estimates described in this paper. Rank orders were assigned by sorting compounds from highest activity to lowest activity based on (from left to right) their activity against asexual blood stage parasites, infection intensity in the mosquito, oocyst prevalence in the mosquito, normalized oocyst prevalence, and the difference between asexual blood stage activity and normalized oocyst prevalence.
